# Depression and suicidal risk in autism spectrum disorder: efficacy of intranasal esketamine. Clinical experience from a public mental health service in Italy

**DOI:** 10.1192/j.eurpsy.2025.1204

**Published:** 2025-08-26

**Authors:** A. Guffanti, F. Mazzoni, A. Silva, F. Calorio, P. Politi, N. Brondino, M. Olivola

**Affiliations:** 1 Dipartimento di Scienze del Sistema Nervoso e del Comportamento, Università degli Studi di Pavia; 2 Dipartimento di Salute Mentale e Dipendenze, ASST di Pavia, Pavia; 3 Dipartimento di Salute Mentale, ASST Fatebenefratelli-Sacco, Milano, Italy

## Abstract

**Introduction:**

Depressive disorders and anxiety disorders are typical comorbidities in autism spectrum disorder (ASD), with a reported prevalence of 20% and 9%, respectively (Lai, 2019). Autistic subjects are more at risk for suicidal thoughts and behaviors compared to typically developing peers; moreover, depressive symptoms are often resistant to both pharmacological and psychotherapeutic treatments. In 2019 intranasal esketamine was approved in Italy for treatment-resistent depression (TRD). We observed two young outpatients affected by autism (diagnosis made by “Lab-Aut”, a specialized mental health service in Pavia) treated with esketamine in our ambulatory for TRD. Clinical information and personal details were summarized in **Image 1**.

**Objectives:**

To evaluate the clinical response to intranasal esketamine in subjects with major depression in ASD and to compare the outcome with neurotypical patients.

**Methods:**

Autism diagnosis wase made according to ADOS-2 and ADI-R scores, confirmed by a clinical judgment of senior psychiatrist. Our follow-up protocol during esketamine treatment consists in the following scales: MADRS, C-SSRS, DES-II, Psychace Scale, Reading the Mind in the Eyes Test, HAM-A, HAM-D, BDI, PANSS. Psychometric evaluation was performed at T0 (before esketamine), T1 (one week of pharmacotherapy), T2 (one month), T3 (2 months) T4 (3 months) and T5 (6 months). We collected results from neurotypical patients (n=12) and autistic patients (n=2) between 2022 and 2024.

**Results:**

Both in autistic patients and in neurotypical ones we noticed a premature decrease in depressive symptoms and a reduction of suicidal thoughts. This improvement was testified by a reduction from T0 to T1 in MADRS total score and in C-SSRS sub-score focused on intensity of suicidal ideation. The reduction was maintained during the observation period **(Image 2)**. Due to the small sample size of autistic patients, we couldn’t reach the statistical significance threshold for this population. Considering the entire sample (n=14) we obtained significant results (T0-T1: MADRS decrease p=0,00006, C-SSRS decrease p=0,00009. T0-T5 MADRS and C-SSRS variations p<0,00001). Comparing our sub-samples, it’s possible to notice a similar trend in follow-up between autistic and neurotypical patients. **(Image 3)**.

**Image 1:**

**Figure:**
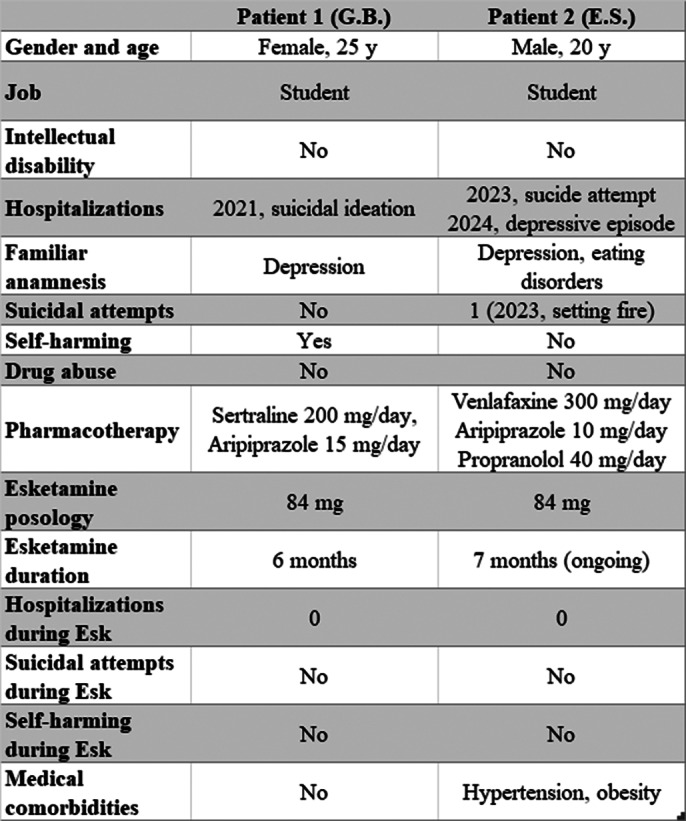

**Image 2:**

**Figure:**
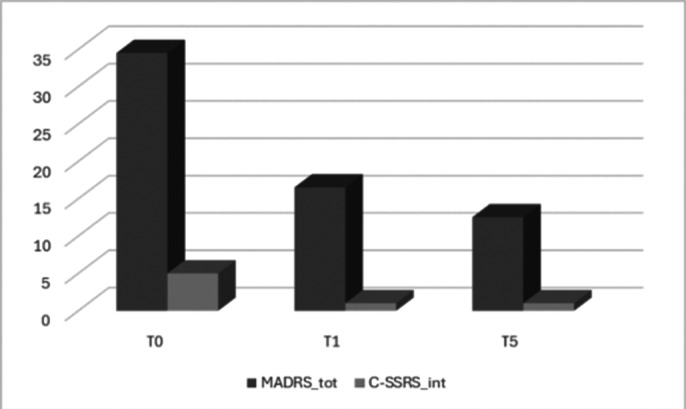

**Image 3:**

**Figure:**
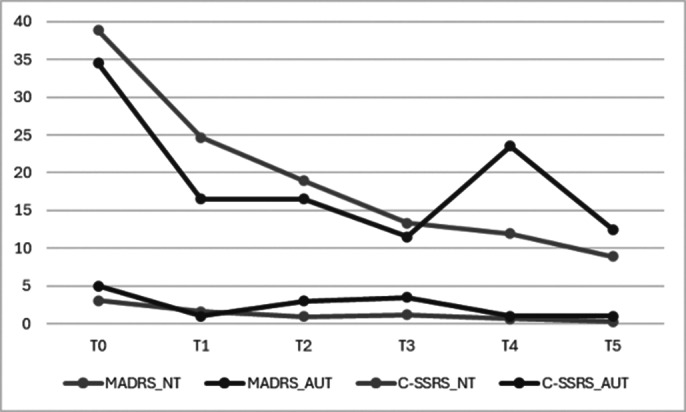

**Conclusions:**

Our preliminary data coming from clinical experience suggest the efficacy of intranasal esketamine in depressive episodes occurring in autistic patients. A larger sample size of autistic patients will be necessary to set a comparative study and to give significance to these results. Esketamine could represent an important therapeutic option in depressed patients with ASD comorbidity.

**Disclosure of Interest:**

None Declared

